# Pelvic rotation parameters related to in-brace correction in patients with idiopathic scoliosis

**DOI:** 10.1186/s40001-020-00437-y

**Published:** 2020-09-17

**Authors:** Kepeng Li, Jun Miao, Jingan Zhang

**Affiliations:** 1 Clinical Department of Orthopaedics, Tianjin Medical University, 406 Jiefang South Road, Hexi District, Tianjin, China; 2 Spine Surgery, Tianjin Hospital, 406 Jiefang South Road, Hexi District, Tianjin, China; 3 Tianjin Hospital, 406 Jiefang South Road, Hexi District, Tianjin, China

## Abstract

**Background:**

To identify the pelvic parameters affecting in-brace correction (IBC) in patients with idiopathic scoliosis (IS).

**Methods:**

Patients with IS receiving Chêneau brace treatment in our scoliosis center from January 2019 to November 2019 were retrospectively analyzed. Pelvic rotation parameters, including pelvic incidence (PI), sacral slope (SS), pelvic tilt (PT), L/R ratio, were collected. Other radiographic data, such as Risser sign, coronal and sagittal balance, curve location, kyphosis, lordosis of each patient were also recorded to analyze their correlations with IBC. Correlation analyses were performed to identify the classified variables influencing IBC. The principal component analysis was used to extract common factors of radiographic parameters to eliminate interaction effects. The linear regression equation was established using principal components, the variables influencing IBC were identified.

**Results:**

A cohort of 44 patients with IS (36 girls and 8 boys) were included in the present study. The mean IBC was 49.87% (range, 3%–100%). IBC of lumbar IS was negatively correlated with apical rotate factor (ARF, B = –0.385), mainly consisted of pelvic coronal plane rotation (PCPR, 0.449), Cobb angle (CA, 0.575), apical vertebral rotation (AVR, 0.918), and pelvic rotate factor (PRF, B = –0.387), mainly consisted of PT (0.861), PI (0.728), PCPR (–0.570). The regression equation of lumbar IS had statistical significance (*F* = 6.500, *P* = 0.005, R2 = 0.317), whereas statistically significance was not found in the regression equation of thoracic IS (*F* = 2.913, *P* = 0.106). The remaining parameters were not related to IBC.

**Conclusions:**

For lumbar IS, ARF and PRF have negative effects on IBC, coronal and sagittal rotation of the pelvis is related to IBC.

## Background

Idiopathic scoliosis (IS) is a complex 3-dimensional deformity of the spine and pelvis [1]. Different aspects of the interaction between the spine and pelvis were investigated in IS [2]. The pelvis serves as an intermediate structure linking the spine to the lower extremities. Pelvic rotation parameters (PRP) contribute to the instability of the spine resulting in the development and progression of IS [3].

Brace application has been reported to be an effective approach in treating mild-to-moderate IS [4]. Despite the high rate of bracing success, some patients will still experience bracing failure [5]. Previous studies have found that IBC is an independent predictive factor for curve progression in braced patients with IS [6]. IBC refers to the percentage of improvement in the curve size at the initial brace prescription. Given the significance of IBC, some studies have been performed to find related imaging parameters to predict the IBC [7]. Self-parameters of IS, such as primary Cobb angle and coronal deformity angular ratio, was found to correlate with IBC [8].

However, little information is available about the effect of pelvic rotation parameters (PRP) on IBC. The purpose of the present study was to identify 3-dimensional PRP influencing IBC in patients with IS.

## Methods

### Subjects

The data from patients who had a diagnosis of IS and were treated with a Chêneau brace in our scoliosis center from January 2019 to November 2019 were reviewed. Ethics approval was obtained from Tianjin Hospital, the number of the ethical approval was 2020 Medical Ethics Review 057. The inclusion criteria were: (1) thoracic or lumbar curve with apex below T5; (2) treatment with Chêneau brace; (3) initial age at bracing 6–18 years; (4) no previous treatment for scoliosis.

Patients with a diagnosis of non-idiopathic scoliosis from congenital, neuromuscular or other connective tissue diseases were excluded from the study. The study was approved by the Clinical Research Ethics Committee of the hospital.

### Patient evaluation

The brace was adjusted at weekly intervals to balance the optimal IBC and appropriate pad pressure. The medical records and radiographs after 2 months post-bracing were reviewed. Full-length standing posteroanterior and lateral radiographs were made. The patients stood upright in a relaxed manner with the fingers of both hands placed on the ipsilateral clavicles and the upper arms abducted to approximately 45^°^ from vertical. All imaging parameters were extracted by the third author who was not involved in the treatment of the patients. To evaluate the measuring precision, all measurements were performed twice using Surgimap Spine Software(New York, USA).

**Fig. 1:**
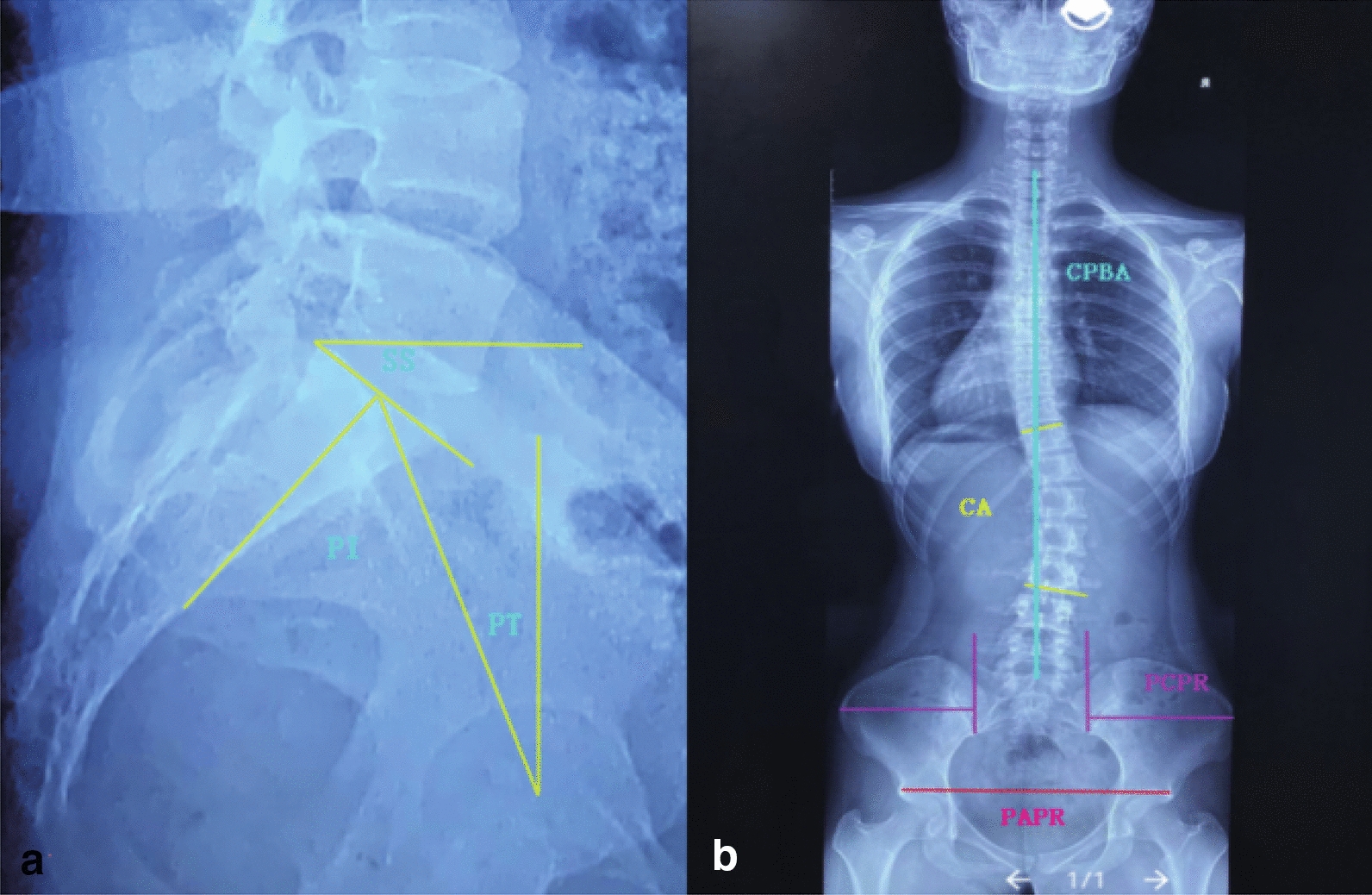
Pelvic rotation parameters measurement

3-Dimensional radiographic parameters of balance and pelvis were measured on the radiograph (Fig. 1).

The definition of PRP parameters and balance parameters:

1. Pelvic axial plane rotation (PAPR) defined as the left/right ratio (L/R ratio) of horizontal distance between the anterior superior iliac spine and the inferior ilium at the sacroiliac joint on the same side.
2. PCPR defined as the angle between the line connecting bilateral eyebrow arch of acetabulum and horizontal line. The angle is positive when the left eyebrow arch is higher than the right.
3. Pelvic incidence (PI) defined as the angle between the perpendicular to the sacral plate and the line joining the midpoint of the sacral plate and the axis of the femoral heads.
4. Sacral slope (SS) defined as the angle between the horizontal line and the sacral plate.
5. Pelvic tilt (PT) defined as the angle between the vertical line and the line joining the midpoint of the sacral plate and the axis of the femoral head.
6. Cobb angle (CA) defined as the angle of the two intersecting lines drawn along the edge of the top and bottom vertebras of the curve. On the top vertebra, the line starts at the high side, is drawn along the top edge and slopes downward according to the angle of the vertebra. Similarly, on the bottom vertebra, the line starts on the low side, is drawn along the bottom edge and will slope in an upward direction.
7. Coronal plane balance angle (CPBA) defined as the angle between the line from the center of the C7 vertebral body to the center of the upper sacral endplate and vertical line.

The relationship between PRP and IBC of thoracic and lumbar IS is described separately. Thoracic IS defined when the apical vertebral located in the thoracic spine, whereas lumbar IS defined when the apical vertebral located in the lumbar spine.

### Statistical analysis

Statistical analysis was performed using SPSS Statistics 25 (IBM Corp, Armonk, New York, USA). The continuous variable was normalized and expressed by the formula:

$$Z\, = \,\left( {X\text{ - }\mu } \right)\text{ / }\sigma ,$$

where x is a specific score, μ is the average, and σ is the standard deviation.

KMO and Bartlett test was used to test the correlation between variables. Dimensions were reduced by principal component analysis and variance maximization rotation. Lithotripsy was used to identify the principal factors, Factors in which eigenvalue was greater than or close to 1 were identified (the larger the eigenvalues, the greater the contribution rate). The screened principal factors were used to create linear regression models. The linear relationship between independent and dependent variables was tested by ANOVA. The goodness of fit was represented by R^2^.

## Results

### Basic characteristics

48 subjects were included initially, but a total of 4 subjects were excluded. In the excluded subjects, 2 of them had too bad image quality to measure, 2 of them had incomplete personal data.

The present study included 44 subjects (36 girls and 8 boys). The mean age was 14.21 ± 2.39 years (range, 6–18), and the mean stage of the Risser sign was 3.40 ± 1.18. The initial major Cobb angle was 28.37 ± 8.07(range, 13.30–44.70), and the initial IBC angle was 15.09 ± 9.66 (range, 0–39.20). The mean IBC rate was 49.87% ± 24.81% (range, 3%–100%).

Among the included patients, 31(72.1%) had lumbar IS, 20 of them were sent to the left and 11 to the right; 12(27.9%) had thoracic IS, all were bent to the right.

### PRP identified to affect in-brace correction

**Fig. 2:**
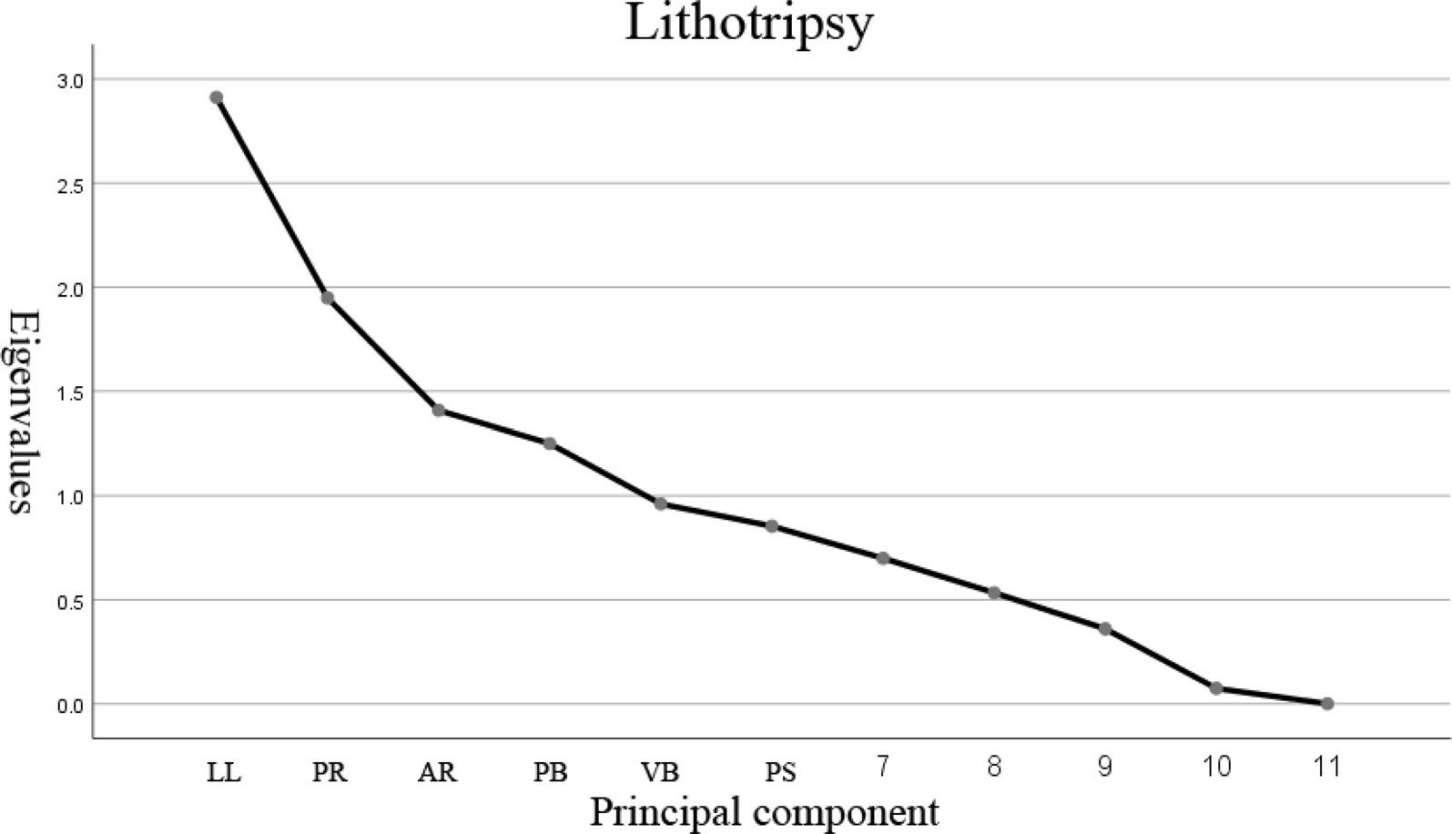
Lithotripsy

As the lithotripsy showed (Fig. 2), the eigenvalues of the principal factors LL, PR, AR, PB were greater than 1, whereas the eigenvalues of the principal factors VB, PS were close to 1. All of the 6 principal factors were identified to be included in multivariate regression analysis (Table 1).

**Table 1:** Factors identified to be included in multivariate regression analysis

Variables	LL	PR	AR	PB	VB	PS
ICA	− .306	.398	.575	.104	− .431	− .131
TKA	.632	− .248	.072	.491	.039	− .236
LLA	.948	.087	− .063	− .023	.077	.047
C7HA	− .050	.177	.109	− .108	.912	− .012
CPBA	− .089	− .025	− .006	.824	− .139	.134
PI	.643	.728	.040	− .159	.015	− .040
SS	.881	.190	− .049	− .162	− .126	.139
PT	.151	.861	.099	− .090	.128	− .174
PAPR	.068	− .179	− .033	.095	.005	.935
PCPR	.172	− .570	.449	− .403	− .184	.087
ARL	− .006	− .014	.918	− .011	.189	− .017

*LL* lumber lordosis factor, *PR* pelvic rotate factor, *AR* apical rotate factor, *PB* coronal balance factor, *VB* vertical balance factor, *PS* pelvic symmetry factor

**Fig. 3:**
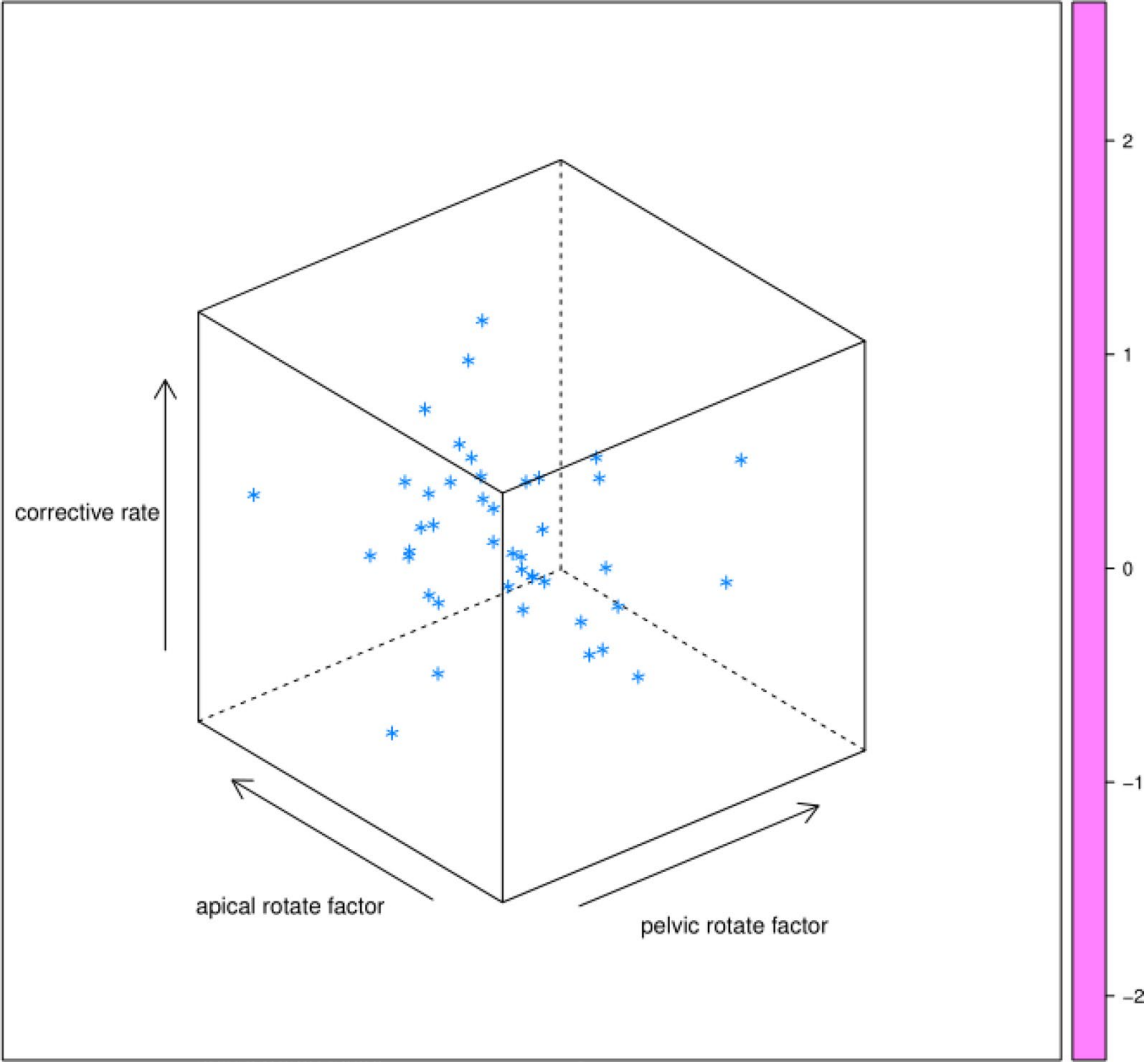
Two factors influencing IBC

Two of the six principal factors (AR, PR) had statistical significance. IBC of lumbar IS was negatively correlated with apical rotate factor (ARF, *B* = −0.385), mainly consisted of pelvic coronal plane rotation (PCPR, 0.449), Cobb angle (CA,0.575), apical vertebral rotation (AVR, 0.918), and pelvic rotate factor (PRF, *B* = −0.387), mainly consisted of PT (0.861), PI (0.728), PCPR (−0.570) The regression equation of lumbar IS had statistical significance (*F* = 6.500, *P* = 0.005, *R*^2^ = 0.317) (Fig. 3), whereas statistical significance was not found in the regression equation of thoracic IS (*F* = 2.913, *P* = 0.106). The remaining parameters were not related to IBC.

Linear regression equation was expressed as: IBC_L_ = 0.47PCPR-1.02PI-1.20PT -0.98CA-0.67AVR.

## Discussion

Evidence has shown that brace treatment can change the natural history of IS [9, 10]. IBC is one of the main predictors for the outcome of brace treatment for IS [6]. It was reported that imaging parameters, such as CA, AVR, were associated with IBC [8, 10]. The same finding was observed in our study. Besides, we analyzed the relationship between PRP and IBC. The main finding from the present study was that coronal and sagittal rotation of the pelvis can influence the IBC of lumbar IS. IBC of lumbar IS was negatively correlated with ARF (mainly consisted of PCPR, CA, AVR) and PRF (mainly consisted of PT, PI, PCPR).

Because of the interaction between the three-dimensional measurement parameters of the spine and pelvis, the main factor analysis was used to reduce the dimension of the original data and to transform many original indexes into a few main factors. So the variability of the original data was represented by the main factors and the interference between the original indexes was reduced. Lithotripsy was used to assess factors accounted for most of the variability in the raw data. Six principal factors that were identified in the study included LL, PR, AR, PB, VB, PS. All of the 6 principal factors were greater than or close to 1. The variability in the raw data was approximately represented by these six factors, then these six factors were included in multivariate regression analysis.

The negative correlation between CA and IBC has been confirmed in our study and previous studies. Studies also revealed that sagittal or coronal imbalance of the spine can decrease IBC and suggested that IS is a complex 3-dimensional deformity of the spine and that sagittal imbalance can affect the curve correction in the coronal plane [7, 11]. Georges and Pierre et al. made the same conclusion that pelvis morphology and standing balance parameters are correlated with CA, they suggested that IS is not only a question of trunk morphology distortion by itself, but is also related to pelvis asymmetrical bone growth and standing neuromuscular imbalance [12]. The same finding was observed in our study.

Pelvis is a necessary base of bracing to correct IS [13]. As a three-dimensional (3D) deformation of the spine, IS does not only influence the spine, but also appears to be caudally extended as pelvic abnormalities were often observed in IS [14]. Individual PRP is following the regional parameters of IS [15]. Therefore, the pre-bracing evaluation of IS should include the regional parameters as well as PRP (Additional file 1).

The three-dimensional nature of IS necessitates a tridimensional assessment and management [16]. To achieve a satisfactory IBC, multiple sets of “three-point force” were used to correct deformities from three-dimensional space [15]. As one of the stress points, the pelvis plays an important role in the IBC of lumbar IS. Reaching a satisfactory IBC is more difficult in cases with higher pre-brace pelvic posterior rotation (PPR). Satisfied lumbar IBC needs the interaction of the spine and pelvis. PPR is a vital compensational mechanism for spinal balance [17]. In IS patients with higher pre-brace PPR, the compensation space of pelvis becomes small. IBC purely comes from the local improvement of spinal curves.

Saba Pasha reported that, in 79% of the TL/L AIS, the pelvis was rotated toward the convex side of the curve in the coronal plane [18]. In our study, 20 of 31 lumbar IS patients had curves bent to the left, and left rotation of the pelvis in the coronal plane is related to a high IBC. It seems that the pelvis rotated toward the convex side of the curve facilitates IBC [19].

Analysis of PRP showed that it influenced the patients’ IBC. PRP should be included in the planning and evaluation of bracing treatment. Indeed, PRP measurement could play a role in treatment outcome and, considering the present analysis, help improve brace action on the IS correction.

The present study had several limitations. First, as a retrospective study, some inherent biases existed. Second, only the patients with Chêneau brace were studied, the applicability of conclusions to patients treated with other types of braces needs further study.

## Conclusions

Our results showed that IBC of lumbar IS can be influenced by the coronal and sagittal rotation of the pelvis. The present study has provided some useful parameters regarding brace design and fabrication. Pre-bracing evaluation of IS should include the regional parameters as well as PRP.

## Supplementary information

**Additional file 1.** Data of patients with IS included in study.

## Data Availability

Data available within the article or its supplementary materials.

## Abbreviations

IBC: In-brace correction
IS: Idiopathic scoliosis
PI: Pelvic incidence
SS: Sacral slope
PT: Pelvic tilt
ARF: Apical rotate factor
PCPR: Pelvic coronal plane rotation
CA: Cobb angle
AVR: Apical vertebral rotation
PRF: Pelvic rotate factor
PAPR: Pelvic axial plane rotation
CPBA: Coronal plane balance angle
